# Molecular Epidemiology of Staphylococcus aureus in the General Population in Northeast Germany: Results of the Study of Health in Pomerania (SHIP-TREND-0)

**DOI:** 10.1128/JCM.00312-16

**Published:** 2016-10-24

**Authors:** Silva Holtfreter, Dorothee Grumann, Veronika Balau, Annette Barwich, Julia Kolata, André Goehler, Stefan Weiss, Birte Holtfreter, Stephanie S. Bauerfeind, Paula Döring, Erika Friebe, Nicole Haasler, Kristin Henselin, Katrin Kühn, Sophie Nowotny, Dörte Radke, Katrin Schulz, Sebastian R. Schulz, Patricia Trübe, Chi Hai Vu, Birgit Walther, Susanne Westphal, Christiane Cuny, Wolfgang Witte, Henry Völzke, Hans Jörgen Grabe, Thomas Kocher, Ivo Steinmetz, Barbara M. Bröker

**Affiliations:** a Department of Immunology, University Medicine Greifswald, Greifswald, Germany; b Friedrich-Loeffler Institute for Medical Microbiology, Ernst-Moritz-Arndt-University of Greifswald, Greifswald, Germany; c Department of Functional Genomics, Interfaculty Institute for Genetics and Functional Genomics, Ernst-Moritz-Arndt-University of Greifswald, Greifswald, Germany; d Department of Restorative Dentistry, Periodontology, Endodontology and Pedodontics, University Medicine Greifswald, Greifswald, Germany; e Institute for Community Medicine, University Medicine Greifswald, Greifswald, Germany; f Centre for Infection Medicine, Institute of Microbiology and Epizootics, Freie Universität Berlin, Berlin, Germany; g Robert Koch Institute, Wernigerode Branch, National Reference Center for Staphylococci, Wernigerode, Germany; h Department of Psychiatry und Psychotherapy, University Medicine Greifswald, Greifswald, Germany; Boston Children's Hospital

## Abstract

Population-based studies on Staphylococcus aureus nasal colonization are scarce. We examined the prevalence, resistance, and molecular diversity of S. aureus in the general population in Northeast Germany. Nasal swabs were obtained from 3,891 adults in the large-scale population-based Study of Health in Pomerania (SHIP-TREND). Isolates were characterized using *spa* genotyping, as well as antibiotic resistance and virulence gene profiling. We observed an S. aureus prevalence of 27.2%. Nasal S. aureus carriage was associated with male sex and inversely correlated with age. Methicillin-resistant S. aureus (MRSA) accounted for 0.95% of the colonizing S. aureus strains. MRSA carriage was associated with frequent visits to hospitals, nursing homes, or retirement homes within the previous 24 months. All MRSA strains were resistant to multiple antibiotics. Most MRSA isolates belonged to the pandemic European hospital-acquired MRSA sequence type 22 (HA-MRSA-ST22) lineage. We also detected one livestock-associated MRSA ST398 (LA-MRSA-ST398) isolate, as well as six livestock-associated methicillin-susceptible S. aureus (LA-MSSA) isolates (clonal complex 1 [CC1], CC97, and CC398). *spa* typing revealed a diverse but also highly clonal S. aureus population structure. We identified a total of 357 *spa* types, which were grouped into 30 CCs or sequence types. The major seven CCs (CC30, CC45, CC15, CC8, CC7, CC22, and CC25) included 75% of all isolates. Virulence gene patterns were strongly linked to the clonal background. In conclusion, MSSA and MRSA prevalences and the molecular diversity of S. aureus in Northeast Germany are consistent with those of other European countries. The detection of HA-MRSA and LA-MRSA within the general population indicates possible transmission from hospitals and livestock, respectively, and should be closely monitored.

## INTRODUCTION

Staphylococcus aureus is a common human pathogen that is able to elicit a wide range of infections, including skin and soft tissue infections, toxin-mediated diseases, and pneumonia (1–3). At the same time, around 20% of the population carries S. aureus as a persistent commensal in the nasal cavity, with the remainder being intermittently colonized (4, 5). Colonization predisposes for lymphatic and hematogenous spread and subsequent endogenous infection with the colonizing strain (6).

S. aureus exhibits increasing virulence and resistance to various antibiotics, complicating prevention and treatment of infections (2, 3). As a result, the pathogen has become one of the most common infections acquired in hospitals and the community and one of the most difficult to control (2, 7). In Germany, S. aureus is the second most common cause of hospital-acquired infections (8); 16.7% of these nosocomial infections are caused by hospital-acquired methicillin-resistant S. aureus (HA-MRSA) strains. Community-acquired MRSA (CA-MRSA) strains, which represent a major threat to human health in the United States, are still rare in Germany and Europe (8, 9). However, the recent spillover of so-called livestock-associated MRSA (LA-MRSA) from livestock to humans in areas with intensive farming gives rise to concern. Worryingly, no new classes of antibiotics have been developed over the last 30 years (7). This stresses the need for a detailed knowledge of the pathogens' molecular and epidemiological characteristics as a basis for the development of effective measures for the prevention and cure of S. aureus infections.

The human S. aureus population has a highly clonal structure dominated by a dozen clonal clusters (CCs) (10). The pathogen's genome consists of a core genome (ca. 75%), a core variable genome (ca. 10%), and mobile genetic elements (MGEs) (ca. 15%) (11). The core genome is highly conserved across S. aureus strains and comprises genes associated with central metabolism and other housekeeping functions. The core variable genome is strictly linked to particular clonal lineages and includes regulators of virulence gene expression, e.g., the accessory gene regulator (*agr*), and surface proteins (11).

Patterns of MGEs (e.g., plasmids, phages, and pathogenicity and genomic islands) are highly varied between S. aureus isolates but nevertheless are often associated with particular clonal lineages (11, 12). MGEs carry a variety of resistance and virulence genes, encoding, for example, superantigens (SAgs), exfoliative toxins, and pore-forming toxins.

Despite the high genetic variability, all S. aureus genotypes that efficiently colonize humans are able to induce lethal infections (13). Yet, some clonal lineages, including the community-acquired MRSA (CA-MRSA) clone USA300, appear to be more virulent than others, which may be attributed to newly acquired virulence and fitness genes, altered expression of common virulence determinants, and alterations in protein sequence that increase fitness (9, 14).

While the prevalence and molecular diversity of S. aureus within hospitals are very well documented, population-based studies on S. aureus nasal colonization are scarce. Previous studies were often limited in size or focused on selected population groups (12, 15, 16). However, population data are urgently required, as S. aureus colonization is a major risk factor for subsequent invasive S. aureus infections. Moreover, the spillover of MRSA from hospitals and livestock into the community and the spread of highly virulent community-acquired MRSA warrant in-depth monitoring of S. aureus in the general population.

We here report the prevalence and population structure of S. aureus in the general population in Western Pomerania, Germany, sampled in a large-scale comprehensive population-based study of almost 4,000 subjects, the Study of Health in Pomerania (SHIP-TREND) (17). SHIP-TREND includes functional tests for several organs, blood examinations, a whole-body magnetic resonance imaging (MRI), OMICs analyses of body fluids, extensive questionnaires, as well as nose swabs (17). Nasal S. aureus isolates were characterized using *spa* genotyping, antibiotic resistance profiling, and PCR-based virulence gene detection. Our aims were to (i) determine the prevalence of HA-MRSA and CA-MRSA in a large representative sample of the general population, (ii) identify risk factors for S. aureus and MRSA colonization, and (iii) characterize the prevalence, population structure, and molecular characteristics of S. aureus.

## MATERIALS AND METHODS

### Study design.

The Study of Health in Pomerania (SHIP-TREND) is a population-based study in western Pomerania in the Northeast of Germany (17). A stratified random sample of 10,000 adults age 20 to 79 years was drawn from population registries. Sample selection was facilitated by the centralization of local population registries in the Federal State of Mecklenburg/West Pomerania. Stratification variables were age, sex, and city/county of residence. After the exclusion of migrated (*n* = 851) and deceased (*n* = 323) persons, the net sample included 8,826 persons. Because of several reasons (241 did not answer, and 3,367 refused participation), examinations were conducted in 4,420 participants between 2008 and 2012 (17).

The objective of the study is a general assessment of the population health. Hence, SHIP-TREND is not focused on single diseases or colonization with S. aureus but encompasses a wide range of health-related conditions, the collection of various common risk factors, subclinical and clinical disorders, and diseases with the widest focus possible.

A nose swab was obtained from 3,891 participants (92.2%). A subset of participants was assessed at their home using a restricted set of investigations without nose swabs (*n* = 409). The remainder refused the nose swabs but agreed to perform other parts of the SHIP-TREND examination.

Out of 1,052 S. aureus isolates obtained from the SHIP-TREND-0 cohort, three showed an incomplete data set lacking data on bacterial density and/or resistance. Moreover, 25 isolates were not available for genotype analyses. These 28 data sets were excluded from molecular analyses (e.g., correlation of *spa* type with resistance and virulence gene profiles).

### Ethics statement.

The study protocol was approved on 6 March 2008 by the local ethics committee of the University of Greifswald (registration no. BB39/08), and all participants gave informed written consent.

### Analysis of S. aureus nasal carriage.

Nasal samples were collected by trained examiners from both anterior nares by use of a rayon swab (BBL CultureSwab Liquid Stuart; BD, USA). Swabs were inserted into the nasal vestibule, and the swab was rotated four times. Nose swabs were stored/transported at 4°C in a transportable compressor cooler (Mobicool C40; Waeco) and processed within 12 h after sampling. SHIP examiners were trained in nose swabbing and validated on two occasions in May 2008 and October 2009. All examiners produced comparable results, as confirmed by low intra- and interobserver variability.

S. aureus identification was based on a protocol for semiquantitative S. aureus culture using a phenol red mannitol salt broth for S. aureus enrichment and mannitol salt agar (BD, Heidelberg, Germany) for quantification (see the supplemental material for details) (18). The observed colonization densities ranged from <10 CFU (category 1) to more than 3,000 CFU (category 5) obtained from 300 μl of swab transport medium. In this paper, we only report S. aureus colonization *per se*, while the semiquantitative data will be analyzed elsewhere. All bacterial isolates were stored at −70°C until further analysis.

S. aureus was identified by colony morphology on mannitol salt agar plates (BD, Heidelberg, Germany), coagulase test (Bio-Rad, Munich, Germany), and catalase test (bioMérieux, Nürtingen, Germany). Isolates were cryoconserved using Roti-Store cryovials (Carl-Roth, Karlsruhe, Germany). The identity of S. aureus was confirmed by PCR for the species-specific genes gyrase (*gyr*) and nuclease (*nuc*), as described below.

### DNA isolation.

Bacterial DNA was isolated from overnight cultures according to the manufacturer's protocol for the Qiagen DNeasy blood and tissue kit (Qiagen, Hilden, Germany).

### *spa* genotyping.

*spa* genotyping was performed according to published protocols using the primers spa-1113f and spa-1514r (see Table S1 in the supplemental material) (12, 19). When amplification was unsuccessful, the PCR was repeated using the alternative primers spa-239f, spa-1717r, spa-1084f, spa-1095f, spa-1618r, and spa-1517r (20, 64). The PCR products were purified (NucleoSpin gel and PCR Clean-Up kit; Macherey and Nagel, Düren, Germany) and sequenced using both amplification primers by a commercial supplier (LGC Genomics GmbH, Berlin, Germany, or GATC, Constance, Germany). The forward and reverse sequence chromatograms were analyzed with the Ridom StaphType software version 2.2.1 (Ridom GmbH, Würzburg, Germany). Closely related *spa* types (costs, ≤3) were grouped into *spa*-clonal clusters (*spa* CCs) using the BURP algorithm. Short *spa* types with fewer than five repeats were excluded from the cluster analysis (21). *spa* CCs were allocated to multilocus sequence type (MLST) CCs through the SpaServer database (www.spaserver.ridom.de), experimental assessment of MLST in a subset of samples (see below), and/or the scientific literature (12, 21–24). A total of 21 isolates (2.1%) could not be assigned to a CC or ST because they were classified as *spa* singletons (*n* = 8), had very short *spa* repeat sequences (*n* = 10), were *spa* negative (*n* = 1), or were untypeable due to atypical sequences flanking the *spa* repeat region (*n* = 2).

### MLST.

MLST analysis was performed on a subset of 57 S. aureus isolates, as previously reported (25), and STs were identified using the MLST database http://saureus.mlst.net/. MLST was conducted to validate the classification *spa* type singletons and strains with short *spa* repeat sequences, as well as in case of a mismatch between virulence gene profiles and *spa* CC. Novel MLST alleles and MLST types were integrated into the MLST database (ST2796, ST2815, ST2948, ST2949, ST2964, and ST2965). The MLST ST/CC of an MLST-typed *spa* type was then attributed to all isolates of the same *spa* type and closely related *spa* types.

### Antibiotic resistances.

Antibiotic resistances were determined using the Vitek2 system with AST-P608 and AST-P632 cards (bioMérieux, Nürtingen, Germany). The test comprised antibiotics of all major antibiotic classes, including several antibiotics of last resort: aminoglycoside antibiotics (gentamicin and tobramycin), β-lactam antibiotics (penicillin, cefoxitin, and oxacillin), 4-chinolone/fluorchinolone antibiotics (ciprofloxacin, levofloxacin, and moxifloxacin), glycopeptide antibiotics (teicoplanin and vancomycin), lincosamide antibiotics (clindamycin and inducible clindamycin resistance), and others (tetracycline, erythromycin, fosfomycin, fusidic acid, linezolid, mupirocin, nitrofurantoin, rifampin, and tigecycline). Strains were categorized as susceptible (S), intermediate (I), or resistant (R) based on MICs; EUCAST cutoffs were used as resistance breakpoints and were set according to CLSI guidelines (http://www.clsi.org) using the Vitek2 software.

### Multiplex PCR for detection of virulence and resistance genes.

PCR was used to screen for a total of 25 virulence genes. Multiplex PCRs were applied for the detection of genes for staphylococcal enterotoxins (*sea* to *selu*), toxic shock syndrome toxin 1 (*tst*), exfoliative toxins (*eta* and *etd*), and *agr* groups 1 to 4, as previously reported (12, 23).

Two additional multiplex PCRs (CA-MRSA I and II) were established based on published PCR protocols to characterize community-acquired MRSA strains, i.e., the North American USA300 (ST8) and USA400 (ST1), as well as the European ST80-CA-MRSA (26–28). CA-MRSA I included 16S rRNA (controls for DNA quality), *luk-PV*, *MW756* (targeting the genomic island vSA3 in USA400), gyrase (*gyr*), and methicillin resistance (*mecA*); CA-MRSA II included exfoliative toxin d (*etd*, a marker for European ST80-CA-MRSA), ACME cassette (*arcA*, USA300 marker), *seh* (USA400 marker), thermostable nuclease (*nuc*, S. aureus marker), and MW1409 (a Sa2int phage marker targeting USA400). For an overview on the primers, see Table S1 in the supplemental material. All assays were validated using sequenced or well-characterized bacterial control strains, including S. aureus 8325-4 and Escherichia coli (negative control), S. aureus CMRSA80 (06-00300; *lukPV etd mecA*), S. aureus CMRSA8 (06-01172; *lukPV arcA mecA*), and S. aureus CMSSA1 (05-01290; *lukPV seh*). The positive-control strains were kindly provided by the Robert Koch Institute, Wernigerode, Germany. The multiplex PCRs were performed with the GoTaq Flexi DNA polymerase (Promega, Mannheim, Germany). Each reaction mixture (25 μl) contained 5 μl of 5× GoTaq reaction buffer, 2.5 μl of deoxynucleoside triphosphates (1 mM; dATP, dCTP, dGTP, and dTTP; Roche Diagnostics, Mannheim, Germany), 5 μl of MgCl_2_ (25 mM), 0.2 μl of polymerase, and 1 μl of template DNA (10 to 20 μg/ml). In addition, CA-MRSA I contained 3.3 μl of water (distilled water DNase/RNase free; Gibco/Invitrogen) and the following primers (all 5 μM): *16S rRNA*, 0.5 μl; *mecA*, 1 μl; *gyr*, 1 μl; *MW756*, 0.75 μl; and *luk-PV*, 0.75 μl. CA-MRSA II contained 0.3 μl of distilled water and the following primers: *MW1409*, 0.75 μl; *seh*, 2 μl; *arcA*, 0.75 μl; *etd*, 1 μl; and *nuc*, 1 μl. An initial denaturation of DNA at 95°C for 5 min was followed by 30 cycles of amplification (95°C for 30 s, 60°C for 30 s, and 72°C for 60 s), ending with a final extension phase at 72°C for 7 min (afterwards, storage at 4°C). All PCR products were resolved by electrophoresis in 1.5% agarose gels (1× Tris-borate-EDTA buffer; 10 μl per sample), stained with RedSafe nucleic acid staining solution (INtRON Biotechnology, South Korea), and visualized under UV light.

Strains identified as MRSA in the antibiotic resistance assay that were *mecA* negative (*n* = 3) were tested using the recently described alternative *mecA* and *mecC* primers (see Table S1 in the supplemental material) (29). These singleplex PCR mixtures (25 μl) contained 5 μl of 5× GoTaq reaction buffer, 2.5 μl of deoxynucleoside triphosphates (1 mM), 5 μl of MgCl_2_ (25 mM), 9.3 μl of water, 1 μl of each primer (10 μM), 0.2 μl of polymerase, and 1 μl of template DNA (10 to 20 μg/ml). The cycling conditions were the same as those described above.

Rapid discrimination between the ancestral and the animal subpopulation of CC398 was performed by singleplex PCR for a recently described single nucleotide polymorphism in the SAPIG_2511 locus, according to published protocols (30). Primers hlb f5 (5′GTTGCAACACTTGCATTAGC; positions 787 to 806) and hlb r6 (5′CTTTGATTGGGTAATGAT; positions 1730 to 1712) were used for the detection of the intact *hlb* gene (accession no. X13404).

### Minimum spanning tree.

*spa* types were clustered using the minimum spanning tree (MST) algorithm of the *spa* typing plug-in of the BioNumerics software (version 7.1; Applied Maths, Ghent, Belgium) with default settings. *spa* types represented by fewer than five repeats were excluded, since reliable cluster analysis of short-repeat successions seems to be limited (21).

### Statistics.

For analyses, final sampling weights and the stratification variable were considered. Continuous data were presented as mean ± standard deviation and/or median (25% quantile, 75% quantile). Categorical data were presented as percentages. Prevalences (with standard errors [SE]) of colonization with S. aureus, MRSA, or selected CCs were determined.

To test dependencies between two categorical variables, chi-square tests were applied. The chi-square statistics were corrected for the final sampling weights and were converted into F-statistics (design-based F-test). In case of low expected numbers, an unweighted exact Fisher's test was applied. A *P* value of ≤0.05 was considered statistically significant. Analyses were conducted using Stata/SE 12.0.

## RESULTS

### Characteristics of the SHIP-TREND-0 cohort.

The mean ± standard deviation (SD) age of study participants was 51.1 ± 15.1 years (range, 20 to 82 years). In total, 36.2% of the study participants had been exposed to a hospital environment during the last 24 months, either as a patient, a frequent visitor, or due to their profession (Table 1). In detail, 14.4% of the study participants stayed in a hospital within the previous 12 months, 21.3% frequently visited a hospital, nursing home, retirement home, or hospice during the previous 24 months, and 7% were employed in the medical sector.

**TABLE 1:** Descriptive characteristics of the SHIP-TREND-0 participants (*n* = 3,891)

Variable^*a*^	SHIP-TREND-0^*b*^
Sex of participant	
Female	1,974 (50.7)
Male	1,917 (49.3)
Age (yr)	
20–29	356 (9.2)
30–39	623 (16.0)
40–49	815 (20.9)
50–59	844 (21.7)
60–69	744 (19.1)
70–82	509 (13.1)
Hospital stay during previous 12 mo (*n* = 559)	14.4 (559/3,882)
No. of hospitalizations (mean ± SD)	1.4 ± 0.9
No. of hospitalizations (median [25%, 75% quantiles])	1 (1, 1)
Length of stay (mean ± SD) (days)	10.5 ± 14.5
Length of stay (median [25%, 75% quantiles]) (days)	6 (3, 11)
ICU stay	12.9 (72/559)
≥3 visits in hospital, nursing home, retirement home, hospice during previous 24 mo	21.3 (826/3,882)
Nursing someone who visited a hospital, nursing home, retirement home, hospice during the previous 24 mo	6.0 (231/3,882)
Occupation in medical sector	7.9 (302/3,831)
Occupation in veterinary sector	1.5 (56/3,831)
Any hospital contact^*c*^	33.2 (1,287/3,882)
Any hospital contact OR occupation in medical sector^*c*^	36.8 (1,411/3,830)

a ICU, intensive care unit.

b Data are presented as number (%) or % (number/total number), unless otherwise stated.

c Any hospital contact defined as hospital stay during the previous 12 months; ≥3 visits in hospital, nursing home, retirement home, or hospice during the previous 24 months; or nursing someone who visited a hospital, nursing home, retirement home, or hospice during the previous 24 months.

### Nasal carriage of MSSA and MRSA, prevalence and risk indicators.

**FIG 1:**
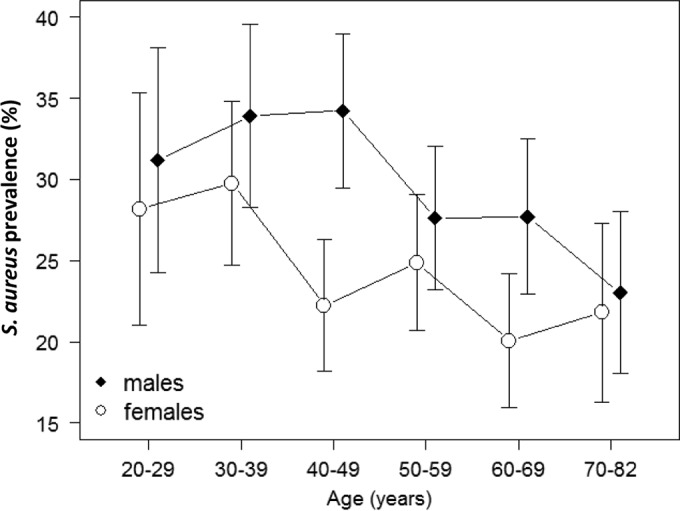
Mean prevalences (error bars show 95% confidence interval) of S. aureus nasal colonization according to age and gender.

The S. aureus prevalence was 27.2%. Nasal S. aureus carriage was more preponderant in males (30.0% in males versus 24.3% in females; *P* < 0.001) and inversely correlated with age (*P* = 0.006; Table 2). If sex and age were considered simultaneously, only the age group 40 to 49 years showed a significantly elevated carriage rate in males versus females (Fig. 1). There was no association between S. aureus carriage and exposure to health care environments.

**TABLE 2:** Prevalence of S. aureus and MRSA in SHIP-TREND-0

Variable	All S. aureus carriage		MRSA carriage	
	% (SE)^*a*^	*P* value^*b*^	% (SE)^*c*^	*P* value^*d*^
Total	27.2 (0.8)		0.34 (0.11)	
Sex				
Female	24.3 (1.0)		0.38 (0.16)	
Male	30.0 (1.1)	**<0.001**	0.30 (0.16)	0.75
Age (yr)				
20–29	29.8 (2.5)		0.57 (0.43)	
30–39	32.0 (1.9)		0 (NA)	
40–49	28.6 (1.6)		0.42 (0.24)	
50–59	26.3 (1.6)		0.41 (0.24)	
60–69	23.8 (1.6)		0 (NA)	
70–82	22.4 (1.9)	**0.006**	0.56 (0.39)	0.18
Hospital stay during previous 12 mo				
No	26.8 (0.8)		0.31 (0.12)	
Yes	29.8 (2.0)	0.17	0.51 (0.36)	0.64
≥3 visits in hospital, nursing home, retirement home, hospice during previous 24 mo				
No	27.6 (0.9)		0.19 (0.09)	
Yes	26.0 (1.6)	0.40	0.88 (0.41)	**0.042**
Nursing someone who visited a hospital, nursing home, retirement home, hospice during previous 24 mo				
No	27.2 (0.8)		0.36 (0.12)	
Yes	28.2 (3.1)	0.74	0 (NA)	1.0
Occupation in medical sector				
No	27.2 (0.8)		0.31 (0.12)	
Yes	27.7 (2.8)	0.83	0.75 (0.53)	0.19
Occupation in veterinary sector				
No	27.2 (0.8)		0.32 (0.11)	
Yes	25.1 (6.0)	0.79	2.00 (1.98)	0.14
Any contact with health care settings^*e*^				
No	27.0 (0.9)		0.22 (0.10)	
Yes	27.6 (1.3)	0.72	0.58 (0.27)	0.32
Any contact with health care settings OR occupation in medical sector^*e*^				
No	27.1 (1.0)		0.14 (0.09)	
Yes	27.4 (1.3)	0.82	0.69 (0.27)	**0.045**

a Prevalence estimates were weighted, and the stratification variable was considered.

b Design-based F-test. *P* values of <0.05 are in bold.

c NA, not applicable.

d Fisher's test, no weighting. *P* values of <0.05 are in bold.

e Any contact with health care settings indicates hospital stay during the previous 12 months; ≥3 visits in hospital, nursing home, retirement home, or hospice during the previous 24 months; nursing someone who visited a hospital, nursing home, or retirement home; or hospice during the previous 24 months.

The prevalence of MRSA in the general population was low (0.34%). MRSA accounted for 0.95% (10/1,052) of the colonizing S. aureus strains. MRSA carriage was associated with frequent visits to hospitals, nursing homes, or retirement homes within the previous 24 months and with being in contact with health care settings either as a patient, visitor, or employee (Table 2). Most of the MRSA carriers (7/10) had been exposed to health care settings within the previous 24 months (Table 3). Moreover, a single person working as an animal caretaker was colonized with LA-MRSA.

**TABLE 3:** Profile of MRSA-positive cases identified in the population-based study SHIP-TREND-0^*a*^

MRSA carrier	MRSA type	Sex	Age (yr)	Hospital stay (last 12 mo)	No. of hospitalizations	Length of stay (days)	Hospitalization in ICU	≥3 visits in hospital, nursing home, retirement home, or hospice (last 24 mo)	Nursing someone who visited a hospital, nursing home, retirement home, or hospice (last 24 mo)	Occupation	Any hospital contact or occupation in medical sector
sh19149	HA-MRSA	Female	28	No	NA	NA	NA	Yes	No	Clerk	Yes
sh18700	HA-MRSA	Female	77	Yes	2	18	No	Yes	No	Accountant	Yes
sh35221	HA-MRSA	Male	28	No	NA	NA	NA	Yes	No	Clerk	Yes
sh08277	HA-MRSA	Male	41	No	NA	NA	NA	No	No	Paramedic	Yes
sh49193	HA-MRSA	Male	47	Yes	8	25	Yes	Yes	No	Road construction worker	Yes
sh48823	HA-MRSA	Female	52	No	NA	NA	NA	Yes	No	Clerk	Yes
sh13413	HA-MRSA	Female	41	No	NA	NA	NA	No	No	Physiotherapist	Yes
sh42507	LA-MRSA	Male	53	No	NA	NA	NA	No	No	Animal caretaker	No
sh19108	HA-MRSA	Female	72	No	NA	NA	NA	No	No	Retired clerk	No
sh12648	HA-MRSA	Female	56	No	NA	NA	NA	No	No	Facility manager	No

a NA, not applicable.

### Most MRSA isolates belong to the pandemic HA-MRSA-ST22 lineage.

Nine out of 10 MRSA isolates represented HA-MRSA lineages endemic to Europe. Most HA-MRSA isolates (*n* = 8) belonged to the pandemic European HA-MRSA-ST22 lineage (Table 4). Moreover, we detected one HA-MRSA-ST5 strain. We also isolated a single LA-MRSA strain (LA-MRSA-ST398) within the SHIP-TREND-0 cohort. Notably, we did not detect any CA-MRSA strains in our sampling cohort.

**TABLE 4:** Genotype, virulence gene profile, and antibiotic resistances of MRSA isolates

Strain ID	Genotype				Virulence gene(s)^*c*^						Resistance gene^*d*^																	
	*spa* type	MLST^*a*^	Deduced MLST CC^*b*^	Endemic European MRSA lineage	*agr*	Non-*egc* SAg	*egc* SAg	*eta*, *etd*	*luk-PV*	*mecA*	CEF	CLI	Ind. CLI	TET	ERY	FOS	FUS	GEN	LVX	LZD	MUP	OXA	PEN	RIF	TEC	TGC	TOB	VAN
sh08277	t010	ND	CC5	ST5 Rhine Hesse MRSA	*2*	a, d, j, r	+	−	−	+	+	−	−	−	−	−	−	−	−	−	−	+	+	−	−	−	+	−
sh19108	t020	ND	CC22	ST22/Barnim MRSA	*1*	−	+	−	−	+	+	+	−	−	+	−	−	−	+	−	−	+	+	−	−	−	−	−
sh12648	t020	ND	CC22	ST22/Barnim MRSA	*1*	−	+	−	−	+	+	−	−	−	−	−	−	−	+	−	−	+	+	−	−	−	−	−
sh48823	t032	ND	CC22	ST22/Barnim MRSA	*1*	c, l	+	−	−	+	+	+	−	−	+	−	−	−	+	−	−	+	+	−	−	−	−	−
sh49193	t032	ND	CC22	ST22/Barnim MRSA	*1*	c, l	+	−	−	+	+	+	−	−	+	−	−	−	+	−	−	+	+	−	−	−	−	−
sh19149	t032	ND	CC22	ST22/Barnim MRSA	*1*	−	+	−	−	+	+	+	−	−	+	−	−	−	+	−	−	+	+	−	−	−	−	−
sh35221	t032	ND	CC22	ST22/Barnim MRSA	*1*	−	+	−	−	+	+	+	−	−	+	−	−	−	+	−	−	+	+	−	−	−	−	−
sh13413	t032	ND	CC22	ST22/Barnim MRSA	*1*	−	+	−	−	+	+	−	−	+	−	−	−	−	+	−	−	+	+	−	−	−	−	−
sh18700	t032	ND	CC22	ST22/Barnim MRSA	*1*	−	+	−	−	+	+	−	−	−	−	−	−	−	+	−	−	+	+	−	−	−	−	−
sh42507	t034	ST398	CC398	ST398 LA-MRSA	*1*	−	−	−	−	+	+	+	−	+	+	−	−	−	−	−	−	+	+	−	−	−	−	−

a ND, not determined.

b *spa* types were clustered by BURP analysis into CCs, and corresponding MLST CCs were deduced using the Ridom database.

c Staphylococcal enterotoxins (SEs) are indicated by single letters (a = *sea*, d = *sed*, j = *sej*, r = *ser*, c = *sec*, l = *sel*). *agr*, accessory gene regulator (*1*, *agr1*; *2*, *agr2*); *egc*, superantigen genes of the enterotoxin gene cluster, i.e., *seg*, *sei*, *sem*, *sen*, *seo*, and *seu*; *eta*, *etd*, exfoliative toxins a and d, respectively; *luk-PV*, Panton-Valentine leukocidin gene; *mecA*, methicillin resistance gene.

d FOX, cefoxitin; CLI, clindamycin; ind. CLI, inducible clindamycin resistance; TET, tetracycline; ERY, erythromycin; FOS, fosfomycin; FUS, fusidic acid; GEN, gentamicin; LVX, levofloxacin; LIN, linezolid; MUP, mupirocin; OXA, oxacillin; PEN, penicillin G; RIF, rifampin; TEC, teicoplanin; TGC, tigecycline; TOB, tobramycin; VAN, vancomycin.

As expected, all HA-MRSA strains were resistant to multiple antibiotics (Table 4; see also Table S2 in the supplemental material). The majority of MRSA strains were resistant to levofloxacin (8/10), clindamycin (6/10), and erythromycin (6/10). Only a minority of isolates were resistant to tobramycin, tetracycline, and co-trimoxazole. No vancomycin resistance was observed. Moreover, all MRSA strains were susceptible to mupirocin, an antibiotic commonly used for sanitizing MRSA carriers (31).

In contrast, the prevalence of antibiotic resistance in MSSA strains was low. Around 62% of the MSSA strains were resistant to β-lactamase-susceptible penicillins (see Table S2 in the supplemental material). Resistances to other antibiotics were rare: between 1 and 6% of MSSA strains were resistant to levofloxacin, clindamycin, erythromycin, and tetracycline. Furthermore, we detected single strains with resistance to gentamicin, co-trimoxazole, and fusidic acid.

### The S. aureus population is highly diverse.

*spa* typing revealed a diverse but also highly clonal S. aureus population structure. We identified a total of 357 *spa* types. The majority of *spa* types (*n* = 248) were represented by single isolates, illustrating the high diversity of the S. aureus population. On the other hand, the 10 most common *spa* types comprised more than one-third (399/1,024 isolates) of all nasal isolates: t012 (*n* = 63), t091 (*n* = 53), t084 (*n* = 52), t008 (*n* = 49), t015 (*n* = 47), t021 (*n* = 41), t005 (*n* = 29), t056 (*n* = 26), t078 (*n* = 23), and t346 (*n* = 16).

**FIG 2:**
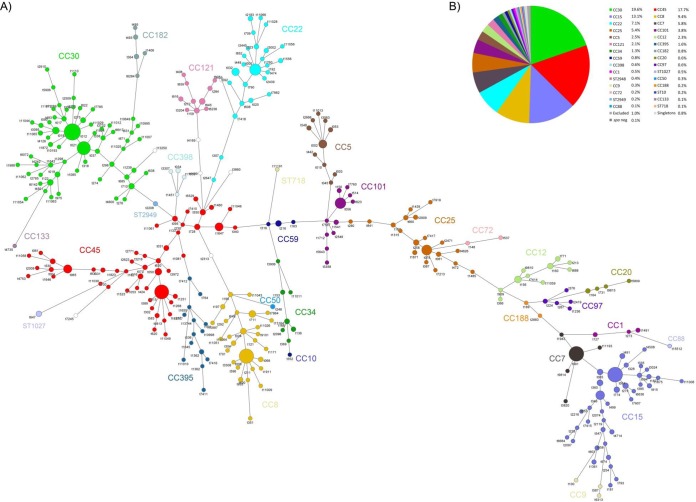
The S. aureus population structure is highly diverse. (A) Minimum spanning tree generated from *spa* data using the BioNumerics software. Each sphere, or node, represents a unique *spa* type. The size of each node indicates the number of S. aureus isolates per *spa* type. The length between two nodes reflects the genetic distance between the two bordering *spa* types (maximum neighbor distance, 1.00). Nodes are color-coded according the presumptively associated clonal cluster. White circles represent singletons, i.e., strains that were not assigned to any CC. The identification of CCs is based on the BURP algorithm, as implemented in the Ridom StaphType software, aided by MLST sequencing of selected strains. (B) Prevalence of CCs and STs within the SHIP-TREND-0 cohort. *spa* types marked as excluded were not assigned to clusters, as their repeat sequence included fewer than 5 repeats, and no reliable information about phylogenetic relatedness can be inferred. *spa* types marked as singletons could not be assigned to a CC. CCs were color-coded in the same manner as in panel A.

The relationship between *spa* types was visualized in a minimum spanning tree (Fig. 2). This graph illustrates the extremely diverse but also highly clonal S. aureus population structure. Closely related *spa* types were assigned to 30 CCs or sequence types (STs) (Fig. 2; see Table S3 in the supplemental material). The most common lineage was CC30, which accounted for 19.6% of the isolates, followed by CC45 (17.7%), CC15 (13.1%), CC8 (9.4%), CC22 (7.1%), CC7 (5.8%), and CC25 (5.4%). These major 7 CCs included 78.0% of all isolates. The largest CCs also showed the highest diversity of *spa* types, suggesting long-term diversification of these lineages (Fig. 2; see Table S3). CC7 forms an exemption, with low *spa* type diversity: 89.8% (53/59) isolates belong to t091, while the remaining 6 isolates belong to 5 different *spa* types (Fig. 1).

### Livestock-associated MRSA and MSSA have spread to the general population.

Within our sampling cohort, 2.1% of the strains belong to CCs associated with both humans and livestock, i.e., CC398 (*n* = 6), CC1 (*n* = 5), CC9 (*n* = 4), and CC97 (*n* = 6), or livestock only, i.e., CC133 (*n* = 1). Among these, there was a single MRSA strain in CC398. LA isolates are frequently resistant to tetracycline (Tet^r^) (32). Moreover, these strains typically lack the immune evasion cluster (IEC), which is located on *Sa3int* phages and encodes human-specific virulence factors (33). The CC398-MRSA group lacked the IEC and was Tet^r^, implying a recent livestock origin (see Table S4 in the supplemental material). Among the CC398-MSSA isolates, only two isolates seemed to be of recent livestock origin (Tet^r^ and either IEC negative or positive for an ORFSAPIG_2511 variant characteristic of the animal population). The other three CC398-MSSA isolates likely represented human-adapted S. aureus strains, because they were tetracycline susceptible (Tet^s^), harbored the IEC, and lacked the respective ORFSAPIG_2511 single nucleotide polymorphism (SNP). Similarly, all CC9 and CC97 isolates were Tet^s^ and encoded the IEC, suggesting long-term adaptation to human hosts. All CC1 isolates were Tet^r^, and some lacked the IEC (*n* = 3). The CC133 isolate was IEC negative and Tet^s^. To conclude, the CC133 isolate as well as a subgroup of the CC398 (*n* = 3) and CC1 isolates (*n* = 3) were probably of recent livestock origin.

### *agr* type and virulence genes are linked to S. aureus lineages.

**FIG 3:**
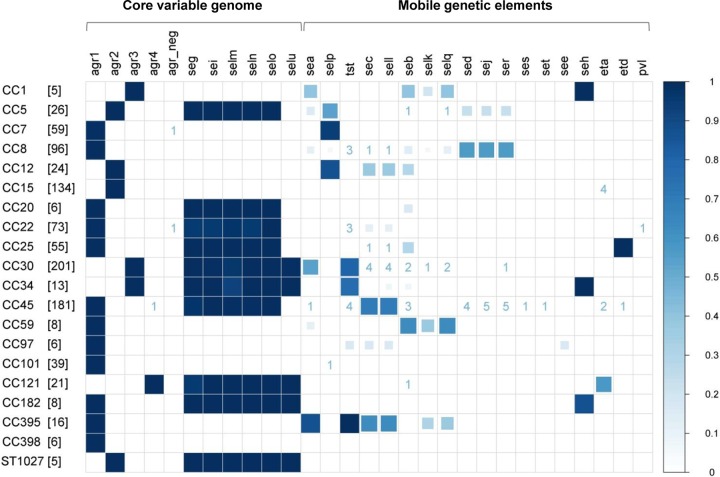
S. aureus virulence genes are linked to CCs. Frequency plot depicting the frequency of virulence genes within each S. aureus CC, as illustrated by both color and size of the squares. If a gene occurred in fewer than 5% of isolates per CC, the number of S. aureus isolates positive for the gene is given. Virulence genes are grouped according to their genomic location. *agr* (*agr1* to -*4*; *agr_neg*, no *agr* detected), and *egc* superantigens (*seg*, *sei*, *sem*, *sen*, *seo*, and *seu*) are core-variable genes. All other virulence genes are located on MGEs. In detail, *sea* and *sep* are encoded by the Sa3int phages, *tst*, *sec*, and *sel* as well as *seb*, *sek*, and *seq* are localized on S. aureus pathogenicity islands, while *sed*, *sej*, *ser*, *ses*, and *set* are carried on plasmids. *eta* and *etd* are located on a phage and plasmid, respectively, while *luk-PV* is located on a phage. The number of isolates per CC is provided in square brackets. CCs with more than 5 isolates are depicted.

The global regulator *agr* (accessory gene regulator) belongs to the core variable genome and is therefore strictly linked to S. aureus lineages (11, 12). As expected, we detected *agr1* in CC7, CC8, CC20, CC22, CC25, CC101, CC45, CC59, CC97, CC133, CC182, CC188, CC395, and CC398; *agr2* in CC5, CC9, CC12, CC15, ST718, and ST1027; *agr3* in CC1, CC30, CC34, and CC88; and *agr4* in CC50 and CC121 (Fig. 3). In three cases (one isolate each from CC22 and CC45, and one isolate of *spa* type t779), no *agr* gene was amplified. A single CC45 isolate (t1081, ST45) carried *agr4* instead of *agr1* (an exception which has been previously reported) (34).

Many virulence genes, such as exfoliative toxins, most superantigen (SAg) genes, and *luk-PV*, encoding the Panton-Valentine leukocidin (PVL), are located on MGEs. Others, e.g., SAg genes of the enterotoxin gene cluster (*egc*), are found in the core variable (CV) genome or the core genome (not in this study). As expected, the CV-carried *egc* SAg genes were very common (60.0% of isolates) and strictly linked to S. aureus lineages (Fig. 3; see Table S5 in the supplemental material). In contrast, virulence genes carried on MGEs were more or less tightly linked to the different CCs. For example, *tst*, which is found on a pathogenicity island, was predominantly found in CC30, CC34, and CC395 with single-*tst*-carrying strains in CC8, CC22, CC45, and CC97. Of note, the *etd* gene, carried on a genomic island, was restricted to CC25.

Each CC possessed a characteristic panel of MGE-encoded virulence factors, with some variations within each CC (Fig. 3) and even within individual *spa* types. For example, all 64 isolates of the predominant *spa* type t012 (CC30) carried the *egc* SAg genes, but only a subset contained the *tst*, *sec*, *sel*, *seb*, and/or *seq* carried on an S. aureus pathogenicity island (SaPI), or the phage-carried *sea* (Fig. 2; and data not shown). Several CCs completely lacked SAg genes: CC15, CC101, CC188, and CC398. Overall, around 22% of the S. aureus isolates were SAg negative. Notably, *luk-PV* was found in two MSSA isolates only: t223 (CC22) and t1445 (ST942). This toxin has been associated with severe skin and soft tissue infections, as well as severe pulmonary infections caused by both MSSA and CA-MRSA (35, 36). *luk-PV*-positive CC22-MSSA isolates were frequently isolated from furunculosis patients in the Szczecin area, which is close to the SHIP study region (36).

We also analyzed whether antibiotic resistances are linked to the genotype. Ampicillin resistance seemed to be lineage associated, as it was generally highly prevalent but rarely occurred in the lineages CC12, CC50, CC97, CC101, CC395, and CC398 (see Fig. S1 in the supplemental material). Moreover, the LA-MSSA lineage CC398 showed a multiresistant phenotype despite being *mecA* negative.

### *spa* typing can misclassify S. aureus isolates with mosaic genomes.

*spa* and MLST are generally highly concordant. However, within this large study cohort, we observed some discrepancies which have been attributed to recombination events involving the *spa* locus (37). For example, CC34 isolates have a mosaic genome with contributions from ST30 and ST10/ST145 (including the *spa* locus) (38). In line with this, MLST, CV, and MGE patterns confirmed a close relationship of CC34 with CC30, while the recombined *spa* genes (t136, t153, t166, and t11011) are characteristic of the ST10 lineage (see Table S6 in the supplemental material). One out of the four t166 isolates and one t352 isolate, however, harbored an unusual CV pattern (*agr2* instead of *agr3*). MLST revealed an ST10 instead of ST34. Thus, both strains likely represent an ancestral ST10 strain without mosaic genome and were hence allocated to CC10 (37, 38).

On the other hand, *spa* types t037 and t710 are generally assigned to ST239, a mosaic strain that has descended from ST8 and ST30 parents but has a *spa* type typical for ST30 strains (http://www.spaserver.ridom.de) (38). The *spa* type t037 and t710 isolates in this study, however, clearly belonged to CC30, based on their CV and MGE gene patterns (see Table S6 in the supplemental material) (12, 22). Both examples illustrate that the allocation of *spa* types to MLSTs without an assessment of characteristic CV or MGE genes can be misleading.

Another peculiarity is *spa* type t605, which consists of two *spa* repeats only (r07-r23). MLST revealed that these isolates belonged to either MLST7 or MLST15, which was also reflected by the typical SAg gene patterns. This suggests at least two different origins of t605 isolates.

### *spa* types are not associated with sex and age of carriers.

S. aureus spa types have been previously associated with sex (t012, t021, t065, and t084) and age (t012) (39). Hence, we tested whether the six most common *spa* types, t012, t091, t084, t008, t015, and t021, were linked to sex or age in our large study cohort. In contrast to the previous report, we did not observe an association of common *spa* types with sex and age (see Table S7 in the supplemental material). Since CV and MGE gene patterns are linked to CCs, we subsequently correlated the most prevalent CCs (>5%, i.e., CC30, CC45, CC15, CC8, CC22, CC7, and CC25) with both factors. The only lineage that showed a moderate association with age was CC8 (major *spa* type t008), gradually declining from 14.6% in age group 20 to 29 years to 5.6% in age group 70 to 82 years (*P* = 0.03) (Table 5). We did not observe an association of S. aureus lineages with sex.

**TABLE 5:** Distribution of the most common CCs (>5% frequency) in S. aureus carriers by sex and age (*n* = 1,013)^*a*^

Variable	CC7		CC8		CC15		CC22		CC25		CC30		CC45	
	% (SE)	*P* value	% (SE)	*P* value	% (SE)	*P* value	% (SE)	*P* value	% (SE)	*P* value	% (SE)	*P* value	% (SE)	*P* value
Total	6.1 (0.8)		9.5 (1.0)		13.3 (1.1)		7.1 (0.8)		5.3 (0.7)		19.7 (1.3)		18.0 (1.3)	
Sex														
Female	7.0 (1.4)		9.7 (1.5)		15.0 (1.8)		7.4 (1.3)		4.8 (1.0)		19.4 (2.0)		18.9 (2.0)	
Male	5.3 (1.0)	0.31	9.3 (1.3)	0.86	11.9 (1.4)	0.18	6.9 (1.1)	0.77	5.6 (1.0)	0.57	19.8 (1.7)	0.87	17.4 (1.7)	0.57
Age (yr)														
20–29	8.2 (3.1)		14.6 (3.6)		11.1 (3.1)		5.5 (2.3)		3.2 (1.9)		15.7 (3.9)		23.3 (4.4)	
30–39	5.4 (1.7)		13.1 (2.6)		11.0 (2.4)		7.9 (2.0)		1.5 (0.8)		21.7 (3.0)		18.8 (2.9)	
40–49	6.7 (1.7)		6.7 (1.6)		15.7 (2.5)		6.3 (1.6)		6.1 (1.7)		18.6 (2.6)		20.0 (2.8)	
50–59	4.7 (1.5)		9.9 (2.1)		9.1 (1.9)		9.9 (2.1)		7.9 (1.9)		22.4 (2.9)		13.1 (2.3)	
60–69	3.7 (1.5)		6.1 (1.8)		20.4 (3.3)		4.9 (1.6)		7.1 (2.0)		15.3 (2.8)		17.2 (3.0)	
70–82	8.0 (2.6)	0.58	5.6 (2.1)	**0.03**	14.8 (3.9)	0.23	7.2 (2.8)	0.44	5.4 (2.2)	0.22	24.3 (4.4)	0.35	15.3 (3.6)	0.22

a Comprises S. aureus isolates with complete data set, excluding isolates which had very short *spa* repeat sequences (*n* = 10), were *spa* negative (*n* = 1), or were untypeable due to atypical sequences flanking the *spa* repeat region (*n* = 2). *P* values are from a design-based F-test. Prevalence estimates were weighted, and design-based variables were considered. *P* values of <0.05 are in bold.

## DISCUSSION

SHIP-TREND-0 is one of the largest studies investigating the prevalence, resistance, and diversity of S. aureus in the general adult population. By combining information on *spa* typing, antibiotic resistance, and virulence genes, this study not only provides new insights into the population structure of S. aureus but will also serve as a reference population for future studies on clinical cohorts.

Compared to other European countries, the prevalence of S. aureus colonization in the German population (27.2%) is in the upper range. den Heijer et al. studied the prevalence of nasal S. aureus carriage in healthy patients across nine European countries and reported an overall crude prevalence of S. aureus nasal carriage of 21.6% (*n* = 6,956), with Hungary (12.1%) and Austria (15.7%) at the lower end and The Netherlands (26.3%) and Sweden (29%) at the upper end (40). Our reported S. aureus prevalence might underestimate the true population prevalence, because high-risk age groups (i.e., children) were excluded, and swabs from body sites other than the nose were not obtained.

We observed 0.34% MRSA prevalence in the general population in Northeast Germany. Mehraj et al. recently reported a higher MRSA prevalence (1.29% [5/389]) in a nonhospitalized population in Braunschweig, central Germany (16). Compared to other European countries, the MRSA prevalence in the Northeast German population was in the upper range. den Heijer et al. reported MRSA prevalences in the healthy community from 0.0% (Sweden) to 0.4% (Belgium) (40). High MRSA prevalences were recently reported from the United States (up to 9.2%) (41).

Our finding that nasal carriage is associated with male sex is in line with several other studies (16, 42–44). Whether this linkage is due to factors other than hormonal disposition is still unclear. We also observed that carriage decreased with advanced age, which confirms previous reports (43, 45). In contrast, we could not reproduce the previously reported association of certain *spa* types with age and gender (39). The long-term persistency of S. aureus carriage in a human subpopulation of ca. 20% suggests a match between certain microbial, environmental, and host factors relevant for the maintenance of colonization. Bacterial factors contributing to successful colonization might involve lineage-specific CV and MGE genes, such as adhesions and immune evasion factors. Understanding of the host genetic susceptibility to S. aureus carriage is still in its infancy. While previous studies have used a candidate gene approach (46–48), the SHIP cohort provides the unique opportunity to identify host gene polymorphisms associated with colonization using a genome-wide association approach.

Risk factors for MRSA carriage in the community are hospitalization history, antibiotic use history, clinic visit history, being a family member of hospital employees, occupational exposure to livestock, and living on a livestock farm (49, 50). In our study, carriage was associated with frequent contacts with health care settings either as a patient, visitor, or employee.

The majority of MRSA strains belonged to the pandemic European HA-MRSA-ST22 lineage (also known as Barnim epidemic strain). ST22 is currently the most common HA-MRSA group in German hospitals (49%) and is spread all over Germany (51). In line with our findings, Tavares et al. reported that the great majority of MRSA strains found in the Portuguese community belonged to clones typically found in the hospitals, in particular, the ST22 clone (52). Moreover, all five MRSA isolates from a population-based study in Braunschweig, Germany, belonged to HA-MRSA-ST22 (16).

The HA-MRSA strains in the SHIP population demonstrated a broad antibiotic resistance profile highly similar to the HA-MRSA strains reported by the German S. aureus Reference Center, with a high incidence of resistance against ciprofloxacin, erythromycin, and clindamycin (51). Notably, we did not detect mupirocin resistance among the 10 MRSA strains, compared to 7% among the HA-MRSA submitted to the German Reference Center in 2014 (53). All of the 10 isolates from our study were susceptible to glycopeptides, linezolid, and tigecycline. As expected, antibiotic resistances in MSSA strains were rare, except for resistance to β-lactamase-susceptible penicillins. Between 4 and 8% of the MSSA strains exhibited erythromycin and clindamycin resistance, possibly due to the wide use of macrolides and lincosamides in the treatment of Gram-positive infections.

We did not find any CA-MRSA strains in our study cohort, suggesting that CA-MRSA is not endemic in Germany. This is also reflected by data from the German National Reference Center for Staphylococci, which reported only 305 cases of CA-MRSA infections in 2014 (predominantly ST5, ST80, ST8, and ST30) (53). To date, epidemiological data on the prevalence of CA-MRSA colonization in the European healthy population are rare. Nevertheless, the burden of CA-MRSA disease seems to vary drastically from country to country. In the United States, community-onset staphylococcal disease is endemic and the major cause for hospital admissions (54). High rates of CA-MRSA colonization (11.4%) in people without risk factors were reported from Portugal (52). The percentage in other European countries, including Germany, Spain, Switzerland, and Norway, was between 0 and 0.4% (39, 55–57). A drawback of SHIP is that only anterior nares were sampled, neglecting other common habitats of S. aureus, such as the perineum, pharynx, or the skin, possibly resulting in an underestimation of the true prevalence of CA-MRSA (9, 40).

The most common S. aureus lineage in our study cohort was CC30 (19.5%), followed by CC45, CC15, CC8, CC22, CC7, and CC25. These patters are in good agreement with a previous study on healthy blood donors from the same region from 2005/2006 (12), suggesting a limited fluctuation of S. aureus lineages over time. Even though the geographical distribution of colonizing S. aureus strains shows some diversity (58), there is pronounced overlap in the dominant CCs. For example, the global success of CC30 is mirrored by the fact that it is the most prevalent lineage in the healthy population in several European countries and the United States, accounting for 20 to 33% of the isolates (12, 39, 56, 58, 59). CC30 is a relatively old and highly successful lineage. *luk-PV*-positive ST30-MSSA strains (known as phage type 80/81) caused a pandemic of S. aureus infections after the Second World War (60). In the course of time, CC30 strains have evolved into major HA- and CA-MRSA clones (9, 60). This points to the ecological success and transmissibility of this CC. Apart from CC30, the lineages CC45, CC15, and CC8 are also frequently found among the five most prevalent lineages in several European studies (39, 42, 57, 58).

Apart from being a human opportunist, S. aureus has long been associated with livestock. Despite the strong interest in LA-MRSA, one has to keep in mind that livestock-associated lineages can be both MSSA and MRSA. Within the SHIP-TREND-0 study, we detected livestock-associated MSSA isolates belonging to CC1, CC398, and CC133. The absence of the IEC and presence of tetracycline resistance suggest a recent animal origin of some of these isolates. Notably, none of the carriers had occupations in the veterinary sector or meat-processing industry.

The discovery of CC398-LA-MRSA boosted interest in livestock as a vessel for the generation of novel MRSA, because people in contact with food production animals are at high risk of colonization with these strains (61). Even though CC398 is by far the most common LA-MRSA lineage in Germany (62), we isolated only a single bona fide CC398-LA-MRSA strain (Tet^r^, IEC negative), which was from an animal caretaker. In contrast, ST398-LA-MRSA represented 23% of all MRSA from hospital screening samples in the Münsterland, a region close to the German-Dutch border. Even though both the Münsterland and the SHIP region western Pomerania are areas with intensive farming, the livestock density in the Münsterland far exceeds that in western Pomerania (530 pigs/km2 versus 39 pigs/km^2^) (63), which might explain the comparably low rate of LA-MRSA in the SHIP cohort.

Virulence gene analyses showed that each S. aureus lineage is characterized by a defined set of core variable and MGE genes. The classification of S. aureus genes into core genome, core variable genome, and MGEs by Lindsay et al. was a milestone in S. aureus molecular epidemiology (11). As expected, core variable genes, i.e., *agr* type and *egc* SAgs, were strictly linked to S. aureus CCs in the SHIP study (11, 12). Moreover, MGE-carried SAg and exfoliative toxin gene patterns typical of different S. aureus lineages were identified, although there was considerable variation in the virulence gene profiles within each S. aureus CC and even within the same *spa* type. Overall, the observed patterns corroborate previous reports (11, 12, 34).

In conclusion, SHIP is one of the largest studies investigating the prevalence, antibiotic resistance, and diversity of S. aureus in the general adult population. By combining information on *spa* typing, antibiotic resistance, and virulence gene repertoire, this study provided insights into the population structure of S. aureus. We showed that S. aureus colonization rates in Northeast Germany are similar to reports from other European countries and that MRSA colonization is still rare. The detection of HA-MRSA and LA-MRSA clones within the general population indicates possible transmission of these strains from the hospitals and livestock, respectively, to the community and warrants close monitoring. In the future, SHIP will serve as a reference population for studies on clinical cohorts. Moreover, we now have the unique possibility to address some long-standing questions in S. aureus research, such as host genetic factors contributing to colonization as well as carriage-associated morbidity and mortality.

## Supplementary Material

Supplemental material
