# Supplementary material for: Molecular Epidemiology of Staphylococcus aureus in the General Population in Northeast Germany: Results of the Study of Health in Pomerania (SHIP-TREND-0)
Source: J Clin Microbiol. 2016 Oct 24;54(11):2774–85. doi: 10.1128/JCM.00312-16 (PMC5078557; doi:10.1128/JCM.00312-16)
Supplement: Supplemental material [file supp_54_11_2774__index.html]

Supplemental material 

# Molecular Epidemiology of Staphylococcus aureus in the General Population in Northeast Germany: Results of the Study of Health in Pomerania (SHIP-TREND-0)

## Supplemental material

- Supplemental file 1 -

  Table S1 (Primers used for multiplex and singleplex PCRs)

  PDF, 117K
- Supplemental file 2 -

  Table S2 (Resistance of MRSA strains to several antibiotics)

  PDF, 75K
- Supplemental file 3 -

  Table S3 (Numbers of *S. aureus* isolates and *spa* types per MLST CC)

  PDF, 127K
- Supplemental file 4 -

  Table S4 (Characterization of putative livestock‐associated *S. aureus* isolates)

  PDF, 142K
- Supplemental file 5 -

  Table S5 (Prevalence of virulence genes in the *S. aureus* isolates)

  PDF, 98K
- Supplemental file 6 -

  Table S6 (Genotypes and virulence gene profiles of strains with putative recombination events)

  PDF, 139K
- Supplemental file 7 -

  Table S7 (Distribution of the six most common *spa* types among *S. aureus* carriers by sex)

  PDF, 129K
- Supplemental file 8 -

  Fig. S1 (*S. aureus* resistances stratified by CCs)

  PDF, 484K
- Supplemental file 9 -

  Semiquantitative analysis of *S. aureus* nasal carriage

  PDF, 22K
