# Supplementary material for: Molecular Epidemiology of Staphylococcus aureus in the General Population in Northeast Germany: Results of the Study of Health in Pomerania (SHIP-TREND-0)
Source: J Clin Microbiol. 2016 Oct 24;54(11):2774–85. doi: 10.1128/JCM.00312-16 (PMC5078557; doi:10.1128/JCM.00312-16)
Supplement: Supplemental material [file JCM.00312-16_zjm999095209so1.pdf]

Table S1. Primers used for multiplex and singleplex PCRs.

PCR	Name	Gene	Primer sequence (5'-3')	Primer size (bp)	Product size (bp)	Reference
Multiplex PCR – CA-MRSA I	16SrRNA-f	*16SrRNA*	AAC TCT GTT ATT AGG GAA GAA CA	23	756	1
	16SrRNA-r		CCA CCT TCC TCC GGT TTG TCA CC	23		1
	Spvl-f	*pvl*	ATC ATT AGG TAA AAT GTC TGG ACA TGA TCC A	31	433	2
	Spvl-r		GCA TCA AST GTA TTG GAT AGC AAA AGC	27		2
	Smw756-f	*MW765*	TGG TTA GCT ATG AAT GTA GTT GC	23	372	3
	Smw756-r		GTC CAT CCT CTG TAA ATT TTG C	22		3
	gyr-f	*gyr*	AGT ACA TCG TCG TAT ACT ATA TGG	24	281	4
	gyr-r		ATC ACG TAA CAG TTC AAG TGT G	22		4
Multiplex PCR – CA-MRSA II	SmecA-f	*mecA*	GTG AAG ATA TAC CAA GTG ATT	21	147	1
	SmecA-r		ATG CGC TAT AGA TTG AAA GGA T	22		1
	Setd-f	*etd*	CCC GTT GAT TAG TCA TGC AG	20	607	2
	Setd-r		TCC AGA ATT TCC CGA CTC AG	20		2
	SarcA-f	*arcA*	GCA GCA GAA TCT ATT ACT GAG CC	23	413	3
	SarcA-r		TGC TAA CTT TTC TAT TGC TTG AGC	24		3
	Sseh-f	*seh*	CAA CTG CTG ATT TAG CTC AG	20	358	2
	Sseh-r		GTC GAA TGA GTA ATC TCT AGG	21		2
	Snuc-f	*nuc*	GCG ATT GAT GGT GAT ACG GTT	21	279	1
	Snuc-r		AGC CAA GCC TTG ACG AAC TAA AGC	24		1
	smw1409-f	*MW1409*	CAA ATT TTG AAA ACT TTA CGC	21	220	3
	smw1409-r		TCC AGG ATT AAA AGA AGC G	19		3
Multiplex PCR - IEC	chp-f	*chp*	TTA GCA ACA ACA GTT TTA GC	20	395	5
	chp-r		TAA GAT GAT TTA GAC TCT CC	20		5
	scn-f	*scn*	ACT TTA GCA ATC GTT TTA GC	20	205	5
	scn-r		CTG AAA TTT TTA TAG TTC GC	20		5
	sak-f	*sak*	CAA AAG ARG TTT ATT ATT TTT AAC TG	26	487	This work
	sak-r		TTA TTT CTT TTC TAT AAY AAC CTT TG	26		This work
	sa3int-f	*Sa3int*	GTT AAA GAA AAT ACC TAC CG	20	144	5
	sa3int-r		TTC TTT WGC GTG TTC TTT TG	20		5
	gyr-f	*gyr*	AGT ACA TCG TCG TAT ACT ATA TGG	24	281	5
	gyr-r		ATC ACG TAA CAG TTC AAG TGT G	22		5
Singleplex PCR	mecA_RKI-f	*mecA*	TGG CTC AGG TAC TGC TAT CCA C	22	776	6
	mecA_RKI-r		AGT TCT GCA GTA CCG GAT TTG C	22		6
	mecLGA251 f	*mecC*	GCT CCT AAT GCT AAT GCA	18	304	6
	mecLGA251 r		TAA GCA ATA ATG ACT ACC	21		6
	2511f	SAPIG_2511	ATGTCAAATACAAATAAAC	19		7
	2511,a1r	SAPIG_2511_animal	GTGAATACAGCTACTAAIT	19	158	7
	2511,h3r	SAPIG_2511_human	GTGAATACAGCTACTAAIC	19	158	7
	hlb f 5	hlb intact	GTTGCAACACTTGCATTAGC	20		This work
	hlb r 6		CTTTGATTGGGTAATGAT	18	944	This work
spa typing	spa-1113f		TAAAGACGATCCTTCGGTGAGC	22	variable	8
	spa-1514r		CAGCAGTAGTGCCGTTTGCTT	21		8
	spa-239f		ACTAGGTGTAGGTATTGCATCTGT	24	variable	9
	spa-1717r		TCCAGCTAATAACGCTGCACCTAA	24		9
	spa-1084f		ACAACGTAACGGCTTCATCC	20	variable	9
	spa-1618r		TTAGCATCTGCATGGTTTGC	20		9
	spa-1095f		AGACGATCCTTCGGTGAGC	19	variable	10
	spa-1517r		GCTTTTGCAATGTCATTTACTG	22		10

1 Zhang K, Sparling J, Chow BL, Elsayed S, Hussain Z, Church DL, Gregson DB, Louie T, Conly JM. 2004. New quadriplex PCR assay for detection of methicillin and mupirocin resistance and simultaneous discrimination of *Staphylococcus aureus* from coagulase-negative staphylococci. J Clin Microbiol 42:4947-4955.

2 Strommenger B, Braulke C, Pasemann B, Schmidt C, Witte W. 2008. Multiplex PCR for rapid detection of *Staphylococcus aureus* isolates suspected to represent community-acquired strains. J Clin Microbiol 46:582-587.

3 Zhang K, McClure JA, Elsayed S, Louie T, Conly JM. 2008. Novel multiplex PCR assay for simultaneous identification of community-associated methicillin-resistant Staphylococcus aureus strains USA300 and USA400 and detection of mecA and Panton-Valentine leukocidin genes, with discrimination of *Staphylococcus aureus* from coagulase-negative staphylococci. J Clin Microbiol 46:1118-1122.

4 Lina G, Piemont Y, Godail-Gamot F, Bes M, Peter M, Gauduchon V, Vandenesch F, Etienne J. 1999. Involvement of Panton-Valentine leukocidin-producing *Staphylococcus aureus* in primary skin infections and pneumonia. Clin Infect Dis 29:1128-1132.

5 van Wamel WJ, Rooijakkers SH, Ruyken M, van Kessel KP, van Strijp JA. 2006. The innate immune modulators staphylococcal complement inhibitor and chemotaxis inhibitory protein of *Staphylococcus aureus* are located on beta-hemolysin-converting bacteriophages. J Bacteriol 188:1310-1315.

6 Cuny C, Layer F, Strommenger B, Witte W.2011. Rare occurrence of methicillin-resistant *Staphylococcus aureus* CC130 with a novel mecA homologue in humans in Germany. PLoS One. 6(9)

7 Cuny C, Abdelbary M, Layer F, Werner G, Witte W. 2015. Prevalence of the immune evasion gene cluster in *Staphylococcus aureus* CC398. Vet Microbiol. 177:1-2.

8 http://www.ridom.de/doc/Ridom_spa_sequencing.pdf

9 Bartels MD, Petersen A, Worning P, Nielsen JB, Larner-Svensson H, Johansen HK, Andersen LP, Jarløv JO, Boye K, Larsen AR, Westh H. 2014. Comparing whole-genome sequencing with Sanger sequencing for *spa* typing of methicillin-resistant *Staphylococcus aureus* . J Clin Microbiol 52:4305-8.

10 Shopsin B, Gomez M, Montgomery SO, Smith DH, Waddington M, Dodge DE, Bost DA, Riehman M, Naidich S, Kreiswirth BN. 1999. Evaluation of Protein A Gene Polymorphic Region DNA Sequencing for Typing of *Staphylococcus aureus* Strains. J Clin Microbiol 37:3556-3563.
