# Supplementary material for: Molecular Epidemiology of Staphylococcus aureus in the General Population in Northeast Germany: Results of the Study of Health in Pomerania (SHIP-TREND-0)
Source: J Clin Microbiol. 2016 Oct 24;54(11):2774–85. doi: 10.1128/JCM.00312-16 (PMC5078557; doi:10.1128/JCM.00312-16)
Supplement: Supplemental material [file JCM.00312-16_zjm999095209so2.pdf]

Table S2. MRSA strains are resistant against a broad range of antibiotics.

Antibiotic class	Antibiotic	% resistant MRSA (absolute No.)[1, 2]	% resistant MSSA (absolute No.)[1, 2]
ß-lactams	Penicillin G	100.0 (10/10)	61.3 (642/1012)
ß-lactamase resistant ß-lactams	Oxacillin	100.0 (10/10)	0.0 (0/1014)
	Cefoxitin	100.0 (10/10)	0.0 (0/978)
Aminoglycosides	Gentamycin	0.0 (0/10)	0.1 (2/1012)
	Tobramycin	10.0 (1/10)	0.1 (2/982)
Glycopeptides	Teicoplanin	0.0 (0/10)	0.0 (0/983)
	Vancomycin	0.0 (0/10)	0.0 (0/1014)
Lincosamides	Clindamycin	60.0 (6/10)	4.3 (43/1009)
	Inducable clindamycin resistance	0.0 (0/10)	5.1 (36/748)
others	Tetracycline	20.0 (2/10)	2.6 (27/1013) [0.2 2/1013)]
	Erythromycin	60.0 (6/10)	6.0 (55/1010) [1.7 (17/1010)]
	Fosfomycin	0.0 (0/10)	0.0 (0/748)
	Fusidic acid	0.0 (0/10)	0.1 (1/748) [0.5 (4/748)]
	Levofloxacin	80.0 (8/10)	2.0 (17/1010) [0.2 (2/980)]
	Linezolid	0.0 (0/10)	0.0 (0/985)
	Mupirocin	0.0 (0/10)	0.0 (0/746)
	Rifampicin	0.0 (0/10)	0.0 (0/985)
	Tigecycline	0.0 (0/10)	0.0 (0/747)

[1] For technical reasons (use of different (updated) Vitek cards) sample size varied.

[2] Numbers in square brackets give prevalence for strains with intermediate resistance.
