# Supplementary material for: Molecular Epidemiology of Staphylococcus aureus in the General Population in Northeast Germany: Results of the Study of Health in Pomerania (SHIP-TREND-0)
Source: J Clin Microbiol. 2016 Oct 24;54(11):2774–85. doi: 10.1128/JCM.00312-16 (PMC5078557; doi:10.1128/JCM.00312-16)
Supplement: Supplemental material [file JCM.00312-16_zjm999095209so3.pdf]

Table S3: Number of *S. aureus* isolates and *spa* types per MLST CC.

MLST-CC	*S. aureus* isolates % (No.)	*spa* types % (No.)
CC1	0.5 (5)	0.8 (3)
CC5	2.5 (26)	3.0 (11)
CC7	5.8 (59)	1.7 (6)
CC8	9.4 (96)	7.8 (28)
CC9	0.3 (3)	0.8 (3)
CC10	0.2 (2)	0.6 (2)
CC12	2.3 (24)	2.8 (10)
CC15	13.1 (134)	10.8 (39)
CC20	0.6 (6)	1.1 (4)
CC22	7.1 (73)	6.9 (25)
CC25	5.4 (55)	6.1 (22)
CC30	19.6 (201)	17.5 (63)
CC34	1.3 (13)	1.9 (7)
CC45	17.7 (181)	15.2 (55)
CC50	0.3 (3)	0.3 (1)
CC59	0.8 (8)	0.8 (3)
CC72	0.2 (2)	0.6 (2)
CC88	0.1 (1)	0.3 (1)
CC97	0.6 (6)	1.4 (5)
CC101	3.8 (39)	3.0 (11)
CC121	2.1 (21)	2.8 (10)
CC133	0.1 (1)	0.3 (1)
CC182	0.8 (8)	1.4 (5)
CC188	0.2 (2)	0.6 (2)
CC395	1.6 (16)	3.6 (13)
CC398	0.6 (6)	1.4 (5)
ST718	0.1 (1)	0.3 (1)
ST1027	0.5 (5)	0.3 (1)
ST2948	0.4 (4)	0.3 (1)
ST2949	0.2 (2)	0.3 (1)
excluded	1.0 (10)	NA (10)
no PCR product	0.1 (1)	NA (1)
nontypable	0.2 (2)	NA (2)
singleton	0.8 (8)	NA (8)
Total	100.0 (1024)	100.0 (361)
