# Supplementary material for: Molecular Epidemiology of Staphylococcus aureus in the General Population in Northeast Germany: Results of the Study of Health in Pomerania (SHIP-TREND-0)
Source: J Clin Microbiol. 2016 Oct 24;54(11):2774–85. doi: 10.1128/JCM.00312-16 (PMC5078557; doi:10.1128/JCM.00312-16)
Supplement: Supplemental material [file JCM.00312-16_zjm999095209so4.pdf]

Table S4: Characterization of putative livestock-associated *S. aureus* isolates

	genotype		virulence genes																			antibiotic resistances																		
strain ID	*spa* type	deduced MLST-CC	*agr* group	non-*egc* SAg	*egc* SAg	*eta, etd*	*luk-PV*	*mecA*	*mecC*	*Sa1int*	*Sa2int*	*Sa3int*	*Sa4int*	*Sa5int*	*Sa6int*	*Sa7int*	*sak*	*chp*	*scn*	intact *hlb* gene	SAPIG_2511 animal population	SAPIG_2511 ancestral population	Cefoxitin	Clindamycin	Inducable Clindamycin Resistace	Tetracycline	Erythromycin	Fosfomycin	Fusidic acid	Gentamycin	Levofloxacin	Linezolid	Mupirocin	Oxacillin	Penicillin G	Rifampicin	Teicoplanin	Tigecycline	Tobramycin	Vancomycin	Occupation
sh14681	t1491	CC1	*3*	*h b*	-	-	-	-	-	-	+	-	-	-	-	-	-	-	-	ND	ND	ND	-	-	ND	-	-	ND	ND	-	-	-	ND	-	-	-	-	ND	-	-	Chief operating officer
sh35374	t273	CC1	*3*	*h*	-	-	-	-	-	-	+	-	-	-	+	-	-	-	-	ND	ND	ND	-	-	-	-	-	-	-	-	-	-	-	-	+	-	-	-	-	-	Gardener
sh36356	t1491	CC1	*3*	*h b*	-	-	-	-	-	-	+	-	-	-	-	-	-	-	-	ND	ND	ND	-	-	-	-	-	-	-	-	-	-	-	-	-	-	-	-	-	-	Hair stylist
sh39012	t127	CC1	*3*	*a h q*	-	-	-	-	-	-	+	+	-	-	-	-	+	-	+	ND	ND	ND	-	-	-	-	-	-	-	-	-	-	-	-	+	-	-	-	-	-	Managing director
sh44629	t127	CC1	*3*	*a h kq*	-	-	-	-	-	-	-	+	-	-	-	-	+	-	+	ND	ND	ND	-	-	-	-	-	-	+	-	-	-	-	-	-	-	-	-	-	-	Printer
sh17518	t4735	CC133	*1*	-	-	-	-	-	-	-	-	-	-	+	-	-	-	-	-	ND	ND	ND	-	+	+	-	+	-	-	-	-	-	-	-	-	-	-	-	-	-	Master mechanic
sh09248	t034	CC398	*1*	-	-	-	-	-	-	-	-	-	-	-	+	-	-	-	-	ND	ND	ND	-	-	ND	+	-	ND	ND	-	-	-	ND	-	+	-	-	ND	-	-	Clerk
sh05143	t3307	CC398	*1*	-	-	-	-	-	-	-	-	+	-	-	-	-	-	+	+	-	-	+	-	-	ND	-	+	ND	ND	-	-	-	ND	-	-	-	-	ND	-	-	Manager
sh25416	t1451	CC398	*1*	-	-	-	-	-	-	+	-	+	-	-	-	-	-	+	+	-	-	+	-	+	+	-	+	-	-	-	-	-	-	-	-	-	-	-	-	-	Lifeguard assistent
sh33199	t11055	CC398	*1*	-	-	-	-	-	-	-	-	+	-	-	+	-	+	+	+	+	+	-	-	-	-	+	-	-	-	-	-	-	-	-	-	-	-	-	-	-	Clerk
sh49730	t11377	CC398	*1*	-	-	-	-	-	-	-	-	+	-	-	-	-	-	+	+	-	-	+	-	+	+	-	+	-	-	-	-	-	-	-	-	-	-	-	-	-	Supervisor, sheltered workshop
sh42507	t034	CC398	*1*	-	-	-	-	*+*	-	+	+	-	-	-	-	-	-	-	-	ND	ND	ND	+	+	-	+	+	-	-	-	-	-	-	+	+	-	-	-	-	-	Animal caretaker
sh07056	t9313	CC9	*2*	-	*gimno*	-	-	-	-	-	+	+	-	-	-	-	+	+	+	ND	ND	ND	-	-	ND	-	+	ND	ND	-	-	-	ND	-	+	-	-	ND	-	-	Surveying technician
sh14554	t209	CC9	*2*	-	*gimno*	*eta*	-	-	-	+	-	+	-	-	-	-	-	+	+	ND	ND	ND	ND	ND	ND	ND	ND	ND	ND	ND	ND	ND	ND	ND	ND	ND	ND	ND	ND	ND	Chief executive officer
sh36402	t100	CC9	*2*	*b*	*gimno*	-	-	-	-	-	-	+	-	-	-	-	+	+	+	ND	ND	ND	-	-	-	-	-	-	-	-	-	-	-	-	+	-	-	-	-	-	Truck driver
sh45810	t587	CC9	*2*	-	*gimno*	-	-	-	-	-	-	+	-	-	-	-	+	+	+	ND	ND	ND	-	-	-	-	+	-	-	-	-	-	-	-	+	-	-	-	-	-	Clerk
sh05633	t224	CC97	*1*	-	-	-	-	-	-	-	-	+	-	-	-	-	+	-	+	-	ND	ND	-	-	ND	-	-	ND	ND	-	-	-	ND	-	-	-	-	ND	-	-	Sales representative
sh08443	t2419	CC97	*1*	*e tst*	-	-	-	-	-	+	-	-	+	-	-	-	+	+	+	-	ND	ND	-	-	ND	-	-	ND	ND	-	-	-	ND	-	-	-	-	ND	-	-	Teacher
sh15070	t376	CC97	*1*	-	-	-	-	-	-	-	-	+	-	-	-	-	+	-	+	-	ND	ND	ND	-	-	-	-	-	-	-	-	-	-	-	-	-	-	-	-	-	Librarian
sh33327	t1236	CC97	*1*	-	-	-	-	-	-	+	-	+	-	-	-	-	+	+	+	-	ND	ND	-	-	-	-	-	-	-	-	-	-	-	-	-	-	-	-	-	-	Clerk
sh38413	t224	CC97	*1*	*cl*	-	-	-	-	-	-	-	-	-	-	-	-	+	-	+	-	ND	ND	-	-	-	-	-	-	-	-	-	-	-	-	+	-	-	-	-	-	Community worker
sh41845	t267	CC97	*1*	-	-	-	-	-	-	-	-	+	-	-	-	-	+	-	+	-	ND	ND	-	-	-	-	-	-	-	-	-	-	-	-	-	-	-	-	-	-	Clerk
