# Supplementary material for: Molecular Epidemiology of Staphylococcus aureus in the General Population in Northeast Germany: Results of the Study of Health in Pomerania (SHIP-TREND-0)
Source: J Clin Microbiol. 2016 Oct 24;54(11):2774–85. doi: 10.1128/JCM.00312-16 (PMC5078557; doi:10.1128/JCM.00312-16)
Supplement: Supplemental material [file JCM.00312-16_zjm999095209so5.pdf]

Table S5. Prevalence of virulence genes in the *S. aureus* isolates.

Gene	%	(No.)
16S rRNA	100.0	(1024)
gyr	100.0	(1024)
nuc	98.5	(1009)
mecA	1.0	(10)
agr1	55.2	(565)
agr2	19.5	(200)
agr3	22.1	(226)
agr4	3.0	(31)
seg	58.5	(599)
sei	59.2	(606)
selm	58.8	(602)
seln	59.2	(606)
selo	60.0	(614)
selu	25.3	(259)
sea	14.3	(146)
selp	9.7	(99)
tst	19.7	(202)
sec	16.0	(164)
sell	16.1	(165)
seb	5.4	(55)
selk	1.6	(16)
selq	3.0	(31)
sed	6.5	(67)
sej	6.6	(68)
ser	6.7	(69)
ses	0.1	(1)
set	0.1	(1)
see	0.1	(1)
seh	2.7	(28)
eta	1.9	(19)
etd	5.6	(57)
luk-PV	0.2	(2)
total	100.0	1024
