# Supplementary material for: Molecular Epidemiology of Staphylococcus aureus in the General Population in Northeast Germany: Results of the Study of Health in Pomerania (SHIP-TREND-0)
Source: J Clin Microbiol. 2016 Oct 24;54(11):2774–85. doi: 10.1128/JCM.00312-16 (PMC5078557; doi:10.1128/JCM.00312-16)
Supplement: Supplemental material [file JCM.00312-16_zjm999095209so6.pdf]

Table S6. Genotype and virulence gene profile of strains with putative recombination events.

strain ID	MRSA phenotype	*spa* type	repeat succession	*spa* CC	MLST	MLST CC (Ridom)[1]	MLST CC[2]	*agr* [3]	*SEs, tst genes* [3]	*egc* [3]	*eta, etd* [3]	*luk-PV* [3]
sh08686	MSSA	t166	04-44-33-31-12-16-34-16-12-25-22-34	CC166	n.d.	CC34	CC34	*3*	*h*	*gimnou*	-	-
sh13812	MSSA	t166	04-44-33-31-12-16-34-16-12-25-22-34	CC166	n.d.	CC34	CC34	*3*	*h tst*	*gimnou*	-	-
sh38343	MSSA	t166	04-44-33-31-12-16-34-16-12-25-22-34	CC166	ST34	CC34	CC34	*3*	*h tst*	*gimnou*	-	-
sh46022	MSSA	t153	04-44-33-31-12-16-34-16-12-33-34	CC166	ST34	CC34	CC34	*3*	*h*	*gimnou*	-	-
sh24254	MSSA	t153	04-44-33-31-12-16-34-16-12-33-34	CC166	ST34	CC34	CC34	*3*	*h tst*	*gimnou*	-	-
sh35663	MSSA	t136	04-44-33-31-12-16-34-16-12-25-22-22-34	CC166	n.d.	CC34	CC34	*3*	*h tst*	*gimnou*	-	-
sh33388	MSSA	t136	04-44-33-31-12-16-34-16-12-25-22-22-34	CC166	n.d.	CC34	CC34	*3*	*h tst*	*gimnou*	-	-
sh19115	MSSA	t136	04-44-33-31-12-16-34-16-12-25-22-22-34	CC166	n.d.	CC34	CC34	*3*	*h*	*gimnou*	-	-
sh18022	MSSA	t136	04-44-33-31-12-16-34-16-12-25-22-22-34	CC166	n.d.	CC34	CC34	*3*	*h tst*	*gimnou*	-	-
sh20389	MSSA	t11011	04-44-31-12-16-34-23-34	Sg	ST34	CC34	CC34	*3*	*h tst*	*gimnou*	-	-
sh17759	MSSA	t166	04-44-33-31-12-16-34-16-12-25-22-34	CC166	ST10	CC34	CC10	*2*	*h cl*	*gimnou*	-	-
sh24658	MSSA	t352	04-54-31-12-16-34-16-12-25-22-34	CC166	ST10	CC34	CC10	*2*	*h*	*gimnou*	-	-
sh24503	MSSA	t037	26-23-25-16-28	CC12	n.d.	ST239	CC30	*3*	*tst*	*gimnou*	-	-
sh12760	MSSA	t037	26-23-25-16-28	CC12	n.d.	ST239	CC30	*3*	*a tst*	*gimnou*	-	-
sh32997	MSSA	t710	15-12-16-02-16-02-24	CC12	n.d.	ST239	CC30	*3*	*a tst*	*gimnou*	-	-
sh05433	MSSA	t710	15-12-16-02-16-02-24	CC12	n.d.	ST239	CC30	*3*	*a tst*	*gimnou*	-	-
sh49604	MSSA	t710	15-12-16-02-16-02-24	CC12	n.d.	ST239	CC30	*3*	*a tst*	*gimnou*	-	-
sh25869	MSSA	t710	15-12-16-02-16-02-24	CC12	n.d.	ST239	CC30	*3*	*tst*	*gimnou*	-	-
sh49815	MSSA	t710	15-12-16-02-16-02-24	CC12	n.d.	ST239	CC30	*3*	-	*gimnou*	-	-
sh08735	MSSA	t605	07-23	excluded	ST7	unkn	CC7	1	*p*	-	-	-
sh44897	MSSA	t605	07-23	excluded	ST7	unkn	CC7	1	*p*	-	-	-
sh23630	MSSA	t605	07-23	excluded	ST15	unkn	CC15	2	-	-	-	-
sh12492	MSSA	t605	07-23	excluded	ST15	unkn	CC15	2	-	-	-	-

1 This MLST-CC was based on data from the Ridom spa-server database.

2 MLST-CC was based on the experimentally determined MLST or CV and MGE gene patterns.

3 Abbreviations: *agr* = accessory gene regulator; Staphylococcal enterotoxins (SEs) are indicated by single letters (a =*sea* , etc.). tst = toxic shock syndrome toxin 1 gene; *egc* = superantigen genes of the enterotoxin gene cluster, i.e. s*eg, sei, sem, sen, seo* , and *seu; eta* /*etd* = exfoliative toxins a and d; *luk-PV* = Panton-Valentine leukocidine gene
