# Supplementary material for: Molecular Epidemiology of Staphylococcus aureus in the General Population in Northeast Germany: Results of the Study of Health in Pomerania (SHIP-TREND-0)
Source: J Clin Microbiol. 2016 Oct 24;54(11):2774–85. doi: 10.1128/JCM.00312-16 (PMC5078557; doi:10.1128/JCM.00312-16)
Supplement: Supplemental material [file JCM.00312-16_zjm999095209so7.pdf]

Table S7. Distribution of the six most common *spa* types among *S. aureus* carriers by sex. N=1013) [1]

		t008		t012		t015		t021		t084		t091	
Variables	Category	% (SE)	p value [2]	% (SE)	p value [2]	% (SE)	p value [2]	% (SE)	p value [2]	% (SE)	p value [2]	% (SE)	p value [2]
Total	-	4.8 (0.7)		5.9 (0.7)		4.1 (0.6)		3.7 (0.6)		4.9 (0.7)		5.4 (0.8)	
Sex	Female	5.4 (1.1)		5.4 (1.1)		4.4 (0.9)		4.4 (1.0)		6.2 (1.2)		6.1 (1.3)	
	Male	4.4 (0.9)	0.48	6.2 (1.0)	0.62	3.9 (0.9)	0.69	3.1 (0.7)	0.27	3.8 (0.8)	0.08	4.8 (1.0)	0.41

1 Comprises *S. aureus* isolates with complete dataset, excluding isolates which had very short *spa* repeat sequences (n=10), were *spa* negative (n=1) or untypable due to atypical sequences flanking the *spa* repeat region (n=2).

2 Design-based F-test. Prevalence estimates were weighted and design-based variables were considered.
