# Supplementary material for: Molecular Epidemiology of Staphylococcus aureus in the General Population in Northeast Germany: Results of the Study of Health in Pomerania (SHIP-TREND-0)
Source: J Clin Microbiol. 2016 Oct 24;54(11):2774–85. doi: 10.1128/JCM.00312-16 (PMC5078557; doi:10.1128/JCM.00312-16)
Supplement: Supplemental material [file JCM.00312-16_zjm999095209so8.pdf]

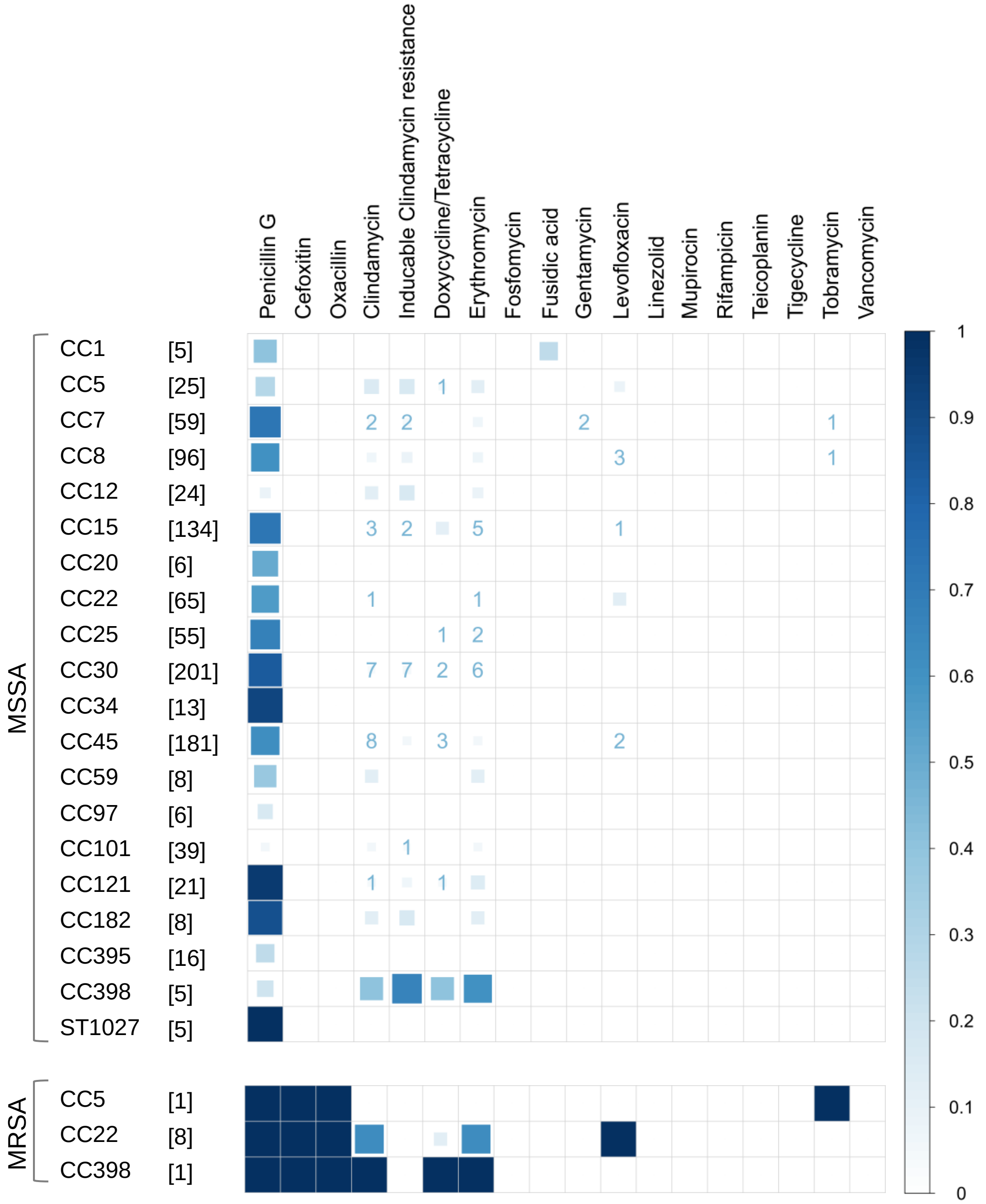

Figure S1. *S. aureus* resistances stratified by CCs. Frequency plot depicting the prevalence of resistances within each *S. aureus* CC for the cross-sectional SHIP cohorts. Antibiotic resistance phenotypes were determined using the Vitek®2 system. The frequency of each resistance phenotype within a CC is illustrated by both colour and size of the square. The number of isolates per CC is provided in square brackets. The upper panel depicts MSSA strains. All CCs with more than 5 isolates are shown. If a resistance occurred in less than 5% of isolates per CC, the number of resistant *S. aureus* isolates is given. The lower panel depicts a frequency plot for MRSA strains. All CCs are depicted (irrespective of the number of isolates).
