# Supplementary material for: Molecular Epidemiology of Staphylococcus aureus in the General Population in Northeast Germany: Results of the Study of Health in Pomerania (SHIP-TREND-0)
Source: J Clin Microbiol. 2016 Oct 24;54(11):2774–85. doi: 10.1128/JCM.00312-16 (PMC5078557; doi:10.1128/JCM.00312-16)
Supplement: Supplemental material [file JCM.00312-16_zjm999095209so9.pdf]

Semiquantitative analysis of *S. aureus* nasal carriage.

Nasal samples were collected by trained examiners from both anterior nares by use of a rayon swab (BBL CultureSwab™ Liquid Stuart, BD, USA). The tubes containing the nasal swab and ca. 500 µl liquid Stuart medium along with a sponge were vortexed for 15 s. The culture swab was then transferred into a falcon tube (15 ml) containing 8 ml phenol red mannitol salt broth for a *S. aureus* enrichment culture (PHM broth, BD, Heidelberg, Germany). The cultivation protocol of the remaining bacterial suspension followed Nouwen et al. with slight modifications (1). For serial dilution of the bacterial suspension mannitol salt agar (MSA, BD, Heidelberg, Germany) culture plates were inoculated with 300 µL and 10 µL of the original bacterial suspension, and with 10 µL and 1 µL of a 1:10 dilution of the suspension. The PHM broth was incubated at 37°C for 7 days; the MSA plates were incubated at 37°C for 48 h and at 25°C for an additional 5 days. After 7 days of incubation, both the PHM broth and the MSA plates were scored. If no *S. aureus* had grown on the MSA plates, but the PHM broth turned yellow (indicating fermentation of mannitol by *S. aureus*), a new MSA plate was inoculated with 10 µL of the PHM broth and incubated as before. The culture results were classified into six categories (0-5). 0: no *S. aureus;* 1: *S. aureus* only in the PHM enrichment culture; 2: *S. aureus* growth additionally on the MSA plate inoculated with 300 µL bacterial suspension (equivalent to 1-30 CFU/300 µl)); 3: *S. aureus* growth on the MSA plate inoculated with 10 µL of the original bacterial suspension (equivalent to 30-299 CFU/300 µl); 4: *S. aureus* growth on the MSA plate inoculated with 10 µL of the diluted bacterial suspension (equivalent to 300-2999 CFU/300 µl); and 5: *S. aureus* growth on all MSA plates, including that inoculated with 1 µL of the diluted bacterial suspension (equivalent to > 3000 CFUs). For a subset of four nose swabs of the SHIP-Trend-0 cohort analyzed after 01.02.2012, semiquantitative data of class 4 and 5 were for technical reasons merged into class 4. Subsequently, all bacterial isolates were stored at -70°C until further analysis.

References

1. Nouwen J, Ott A, Kluytmans-Vandenbergh M, Boelens H, Hofman A, van Belkum A, Verbrugh H. 2004. Predicting the *Staphylococcus aureus* nasal carrier state: derivation and validation of a "culture rule". Clin Infect Dis 39:806-811.
